# Artificial selection for flower size in *Brassica rapa* reveals moderate heritability and many correlated traits

**DOI:** 10.17912/micropub.biology.001691

**Published:** 2025-10-02

**Authors:** Megan L Van Etten

**Affiliations:** 1 Pennsylvania State University, State College, Pennsylvania, United States

## Abstract

With the ultimate goal of better understanding the impact of flower size on pollinator attraction, mating patterns, and plant fitness, four generations of artificial selection were performed on flower diameter in *Brassica rapa* . Selection produced a 90% difference in mean flower size between lines, expanded trait range by 6%, and revealed moderate heritability ( *h* ² = 0.25). It also resulted in correlated responses in flowering time, floral morphology, seed set, and biomass. These divergent lines offer a valuable experimental resource for testing hypotheses about pollinator-mediated selection, ecological trade-offs, and trait integration in plants.

**Figure 1. Results from four generations of artificial selection:**
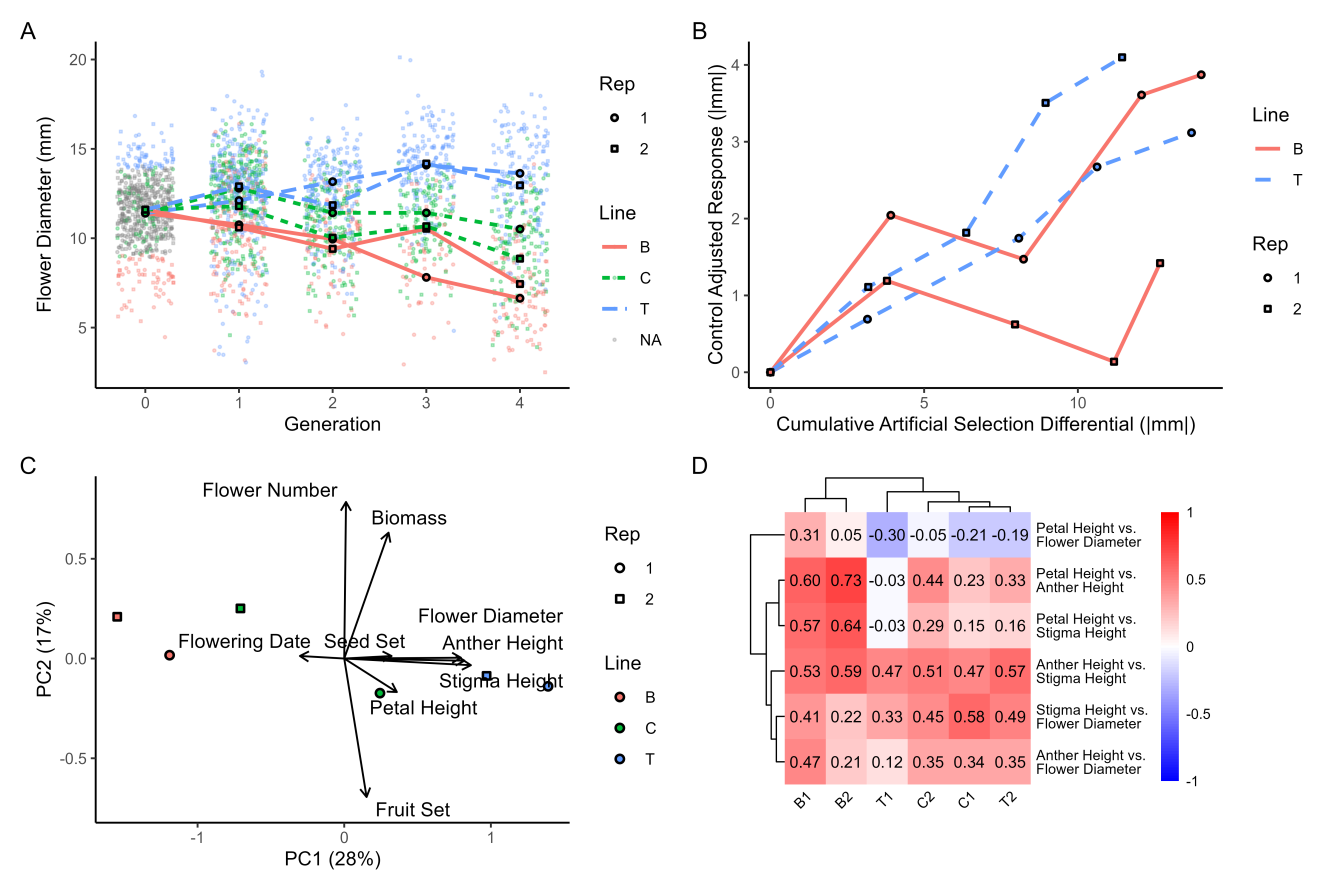
Flower diameter for each line*replicate in each generation (A) and the control-adjusted mean flower diameter (response to selection) as a function of the absolute value of the cumulative artificial selection differential (B). PCA of floral and biomass traits, with line*replicate means (C). Floral size trait correlations per line*replicate (D).

## Description

Flower size is a key trait in plant–pollinator interactions, influencing multiple aspects of reproductive biology. It plays a critical role in attraction; larger flowers tend to be more attractive to pollinators (Conner & Rush, 1996; Galen, 1999; Johnson et al., 1995) and may impact which species visit a given flower (Delgado et al., 2023; Thompson, 2001). In turn, visitor number and identity can shape reproductive success when pollen is limiting or when pollinators differ in their effectiveness (Brunet & Holmquist, 2009; Page et al., 2021; Wolowski et al., 2014), and could even affect the mating system (Gamba & Muchhala, 2023; Herrera, 1987; Matsuki et al., 2008; Rhodes et al., 2017; Santa-Martinez et al., 2021; Wessinger, 2021). Beyond reproduction, flower size can affect the plant's resource budget; larger flowers are typically more costly in terms of nutrients, water, time, and visibility to herbivores (Galen, 1999; Kuppler & Kotowska, 2021; Obeso, 2002). Thus, flower size lies at the intersection many biotic and abiotic interactions. Despite extensive research, many causal relationships between flower size and reproductive outcomes remain unresolved. Experimental manipulation, in particular artificial selection, offers a powerful approach to expand phenotypic variation beyond natural levels, enabling researchers to test specific ecological and evolutionary hypotheses.

In this experiment, artificial selection was used with rapid cycling *Brassica rapa* to increase and decrease floral size (specifically corolla diameter) primarily to develop lines for future studies. *Brassica rapa* is an annual, generalist pollinated, self-incompatible species that has previously undergone artificial selection to accelerate its life cycle (Williams & Hill, 1986), making it amenable for easy and quick usage in experiments. Four generations of selection for the top 10%, bottom 10%, and a random 10% of flower diameter was completed, from which realized heritability was estimated and correlations among other traits determined.

Divergent selection was successful at increasing the mean and range of flower diameter (Fig 1A). At generation zero, flower diameter ranged from 4.46-16.8 mm (mean: 11.5 mm ± 0.06 SE). By generation four the mean flower diameter had increased 15.7% in the top lines (range: 5.56-18.0 mm, mean: 13.3 mm ± 0.20) and decreased 39.1% in the bottom lines (range: 2.5-14.1 mm, mean: 7.00 mm ± 0.21). In both replicates, flower diameter in the control line decreased, likely due to growing conditions. Adjusting for this declining size in the control lines shows a greater response to selection on average in the top lines compared to the bottom lines, primarily due to the low response in replicate 2 of the bottom line (Fig 1B).

The slope of the cumulative selection differential and the response to selection provides an estimate of realized heritability (Fig 1B). Across the four generations, realized heritability was generally moderate (average 0.25) but varied across lines. Replicate 2 in the bottom line had a very low heritability (0.05) while the other replicates were considerably higher and similar (top rep 1 = 0.28, rep 2 = 0.37; bottom rep 1 = 0.25).

After four generations of selection on flower diameter, a variety of other traits were measured to examine trait correlations and indirect selection. Many of the traits measured after four generations of selection were correlated with each other. In a PCA of the measured traits, the first principal component loaded with the flower size traits, seed set, and the inverse of flower date while the second principal component loaded with flower number, biomass, and the inverse of fruit set (Fig 1C). Artificial selection resulted in significant indirect selection on all traits except for fruit set (Table 1). The top selection lines had 3% earlier flowering, 90% larger flower diameter, 12% taller petals, 16% taller anthers, 30% taller stigma, 12% fewer flowers, 51% higher seed set, and 41% higher biomass than the bottom selection lines on average. Interestingly, examining the correlation in floral size traits among replicates and lines showed significant differences (χ ^2^_50_ = 195.92, p < 0.0001). In particular, in the bottom lines, correlations between petal height, anther height, and stigma height were more strongly positively correlated while in the top and C1 lines, petal height and flower diameter were negatively correlated (Fig 1D).

This artificial selection experiment produced divergent lines of *Brassica rapa* that differ in flower size and many correlated traits. These lines represent a valuable tool for studying plant reproduction, floral trait evolution, and trait integration. Moderate realized heritability estimates (~0.25) indicate that sufficient genetic variation was present in the base population to enable short-term evolutionary responses. However, the asymmetrical response to selection, particularly the weak response in one of the bottom lines, suggests limits to continued selection for smaller flower size. Additionally, the bottom lines had lower biomass and from observation often showed other malformations (e.g. malformed leaves, stunted growth, etc), suggesting indirect selection for deleterious alleles associated with reduced growth or vigor. If further selection is needed for smaller flowers, new genetic variation should be introduced.

The experiment also revealed indirect responses in traits related to phenology, reproductive output, and plant architecture. For example, larger-flowered lines flowered earlier and produced fewer flowers with higher seed set and biomass, while smaller-flowered lines tended to have lower biomass but more flowers. These correlated shifts are important for future use of the lines: they may increase realism in studies of pollinator behavior (e.g., examining trait suites rather than single traits), but they may also complicate interpretations if changes in floral size are confounded with changes in plant health and/or resource allocation. Researchers using these lines should consider whether correlated traits are central to their hypotheses or need to be controlled in experimental designs.

Importantly, this study also found that trait correlation structures differed among the selection lines. This suggests that selection can reshape not only mean trait values but also the multivariate integration of floral traits. Such changes have important implications for understanding constraints, modularity, and evolvability. For example, the altered relationships between petal size and organ heights could influence pollinator positioning, contact with reproductive structures, and ultimately plant mating patterns. These lines therefore offer a powerful system for studying how selection acts on integrated floral traits and for exploring broader questions in plant ecology and evolution, including pollinator-mediated selection, floral trait trade-offs, and the evolution of selfing syndromes.

**Table 1.** ANOVAs for each trait, with p-values adjusted using Benjamini-Hochberg correction for multiple tests, and relative pairwise comparisons of line means for traits with significant line effects.

**Table:** 

Trait	Line			Replicate			Line*Replicate			Post-hoc test
	df	F	adj. p-value	df	F	adj. p-value	df	F	adj. p-value	
Flowering Date	2	11.25	<0.0001	1	4.21	0.06	2	0.12	0.92	C=B>T
Flower Diameter	2	222.93	<0.0001	1	8.11	0.009	2	7.78	0.001	T>C>B
Petal Height	2	10.36	0.0001	1	1.35	0.33	2	3.29	0.06	T=C>B
Anther Height	2	70.59	<0.0001	1	13.17	0.0009	2	0.07	0.93	T>C>B
Stigma Height	2	70.58	<0.0001	1	35.19	<0.0001	2	9.12	0.0004	T>C>B
Flower Number	2	3.60	0.048	1	6.01	0.03	2	0.72	0.55	B>C>T
Fruit Set	2	0.80	0.58	1	10.11	0.004	2	0.73	0.55	-
Seed Set	2	22.36	<0.0001	1	1.02	0.40	2	11.99	<0.0001	T>C>B
Biomass	2	3.87	0.039	1	3.51	0.09	2	0.47	0.67	T=C>B

## Methods

Standard Wisconsin Fast Plants® (Carolina Biological Supply Company, Burlington, NC) were planted in 72-well trays for a total of 1008 wells and grown in the greenhouse. Crosses were made between neighboring plants, with each plant acting as a pollen parent for the plant to the right and an ovule parent for the plant on the left. This created 925 families with multiple seeds. One seed from each family was planted as the starting population in either replicate 1 and 2 and supplemented with extra seeds from a previous experiment that was randomly crossed for a total of 500+42 extra seeds (replicate 1) and 425+79 (replicate 2). Plants were grown under artificial lights in a lab setting. For each plant that flowered, corolla diameter at its widest was measured using digital calipers for up to two flowers. Within each replicate, the largest 10% (hereafter “top”), the smallest 10% (“bottom”) and a random 10% (“control”) of the plants were chosen for crosses. Each selected plant was crossed with up to ten other plants using forceps to transfer pollen and oil-based paint pens (Sharpie, Vietnam) to mark the pedicel to track crosses. Seeds were collected and counted.

The same methods were used to continue selection for 3 more generations with the exception of how crosses were determined. To assign crosses, the relatedness between each plant was calculated in R (v4.4.2, R Core Team, 2024) using the pedigreemm package (Bates & Vazquez, 2024). The relatedness matrix was inputted into a custom python script that assigns ten crosses to each plant, calculates the relatedness, and repeats to find the set of crosses that minimize the relatedness.

Realized heritability was calculated as the slope of the response to selection (R) and the cumulative selection (S) for three measures of response (Falconer et al., 1996). R values were calculated for each generation and replicate as:

(1) R _TC_ = Χ _T_ - Χ _C_

(2) R _BC_ = Χ _B_ - Χ _C_

where Χ _T_ , Χ _B_ , and Χ _C_ are the mean flower diameter for the top, bottom and control lines, respectively. The subtraction of the control line controls for any differences due to environment. Selection differentials for each generation and replicate was calculated as:

(3) Σ *_i=1_ (d _i_ - μ) p _i_*

where *d _i_* is the mean flower diameter of the *i* th parent, *μ* is the mean flower diameter of the pool of possible parents, and *p _i_* is the proportion of measured progeny produced by the *i* th parent (Falconer et al., 1996). This method corrects for different contributions between parents to the next generation. The sum of the absolute value of the selection differentials per generation, line, and replicate was used as the cumulative selection (S). A linear model using cumulative S and R was used to determine the slope, which is realized heritability.

The seeds produced from the 4 ^th^ bout of selection (hereafter generation 4) were used to examine traits correlated with flower diameter and indirect selection. All plants were planted in random locations and grown simultaneously. Flowering date was tracked daily. Flower diameter, height (sepal attachment to top of petals), tallest anther height (sepal attachment to top of tallest anther), and stigma height (sepal attachment to top of stigma) were measured using digital calipers for one flower per plant. Crosses within each selection line were performed haphazardly daily to ensure maximum seed and fruit production. After fruit development was complete, the above ground portion of the plant was collected, the number of flowers on the primary inflorescence was counted, all fruits were counted and collected, and the remaining above ground biomass dried and weighed. The seeds from each fruit were counted.

To examine the correlation among traits, a PCA was performed using flowering date, flower diameter, flower height, anther height, stigma height, flower number, fruit set (number of fruits / number of flowers), seed set (number of seeds / number of fruits), and biomass in R using the FactoMineR package (Lê et al., 2008). To examine if the correlation among flower size traits differed among line*replicate, a Box’s M test for homogeneity was performed in R using the biotools package (da Silva et al., 2017). To test for differences in individual traits caused by selection, ANOVAs were performed for each trait with line, replicate, and their interaction as predictors. P-values were adjusted using the Benjamini and Hochberg (1995) correction for multiple tests using the stats package. To compare among lines for each trait, a post-hoc Tukey’s Honest Significant Difference (HSD) test was performed in R using the stats package.

Generative AI (Chat-GPT 4o) was used to draft portions of the analysis code, troubleshoot code, and as a grammar editor of the writing.
